# Supplementary material for: Impact of Diet on Plasma Lipids in Individuals with Heterozygous Familial Hypercholesterolemia: A Systematic Review of Randomized Controlled Nutritional Studies
Source: Nutrients. 2021 Jan 15;13(1):235. doi: 10.3390/nu13010235 (PMC7829745; doi:10.3390/nu13010235)
Supplement: Supplementary file 1 [file nutrients-13-00235-s001.pdf]

Impact of diet on plasma lipids in individuals with heterozygous familial hypercholesterolemia: a systematic review of randomized controlled nutritional studies.

Gabrielle Roy, Anykim Boucher, Patrick Couture, Jean-Philippe Drouin-Chartier

Supplementary material

Table S1: Systematic review search strategy[1]

Database	Step	Search terms	Results
Pubmed	1	“Hyperlipoproteinemia type II” OR “familial hypercholesterolemia” OR “familial hypercholesterolemias”	8,857
	2	"Cholesterol"[Mesh] OR "Cholesterol, LDL"[Mesh] OR "Cholesterol, VLDL"[Mesh] OR "Cholesterol, HDL"[Mesh] OR "Cholesterol, Dietary"[Mesh] OR "Cholesterol Esters"[Mesh] OR "Apolipoproteins"[Mesh] OR "Apolipoproteins B"[Mesh] OR "Apolipoproteins A"[Mesh] OR "Apolipoprotein B-100"[Mesh] OR "Apolipoprotein B-48"[Mesh] OR "Apolipoprotein A-I"[Mesh]	194,890
	3	“clinical trials as topic” [MESH] OR "intervention”[tiab] OR "controlled trial"[tiab] OR "randomized"[tiab] OR "randomised"[tiab] OR "random"[tiab] OR "randomly"[tiab] OR "placebo"[tiab] OR "assignment"[tiab]	1,975,487
	4	#1 AND #2	4,010
	5	#1 AND #2 AND #3	**553**
Web of science	1	((TS=(("Hyperlipoproteinemia type II" OR "familial hypercholesterolemia" OR "Familial hypercholesterolemias") AND ("Cholesterol" OR "Cholesterol, LDL" OR "Cholesterol, VLDL" OR "Cholesterol, HDL" OR "Cholesterol, Dietary" OR "Cholesterol Esters" OR "Apolipoproteins"OR "Apolipoproteins B" OR "Apolipoproteins A" OR "Apolipoprotein B-100" OR "Apolipoprotein B-48" OR "Apolipoprotein A-I") AND ("Intervention Studies" OR "clinical controlled trial" OR "intervention" OR "controlled trial" OR "randomized" OR "randomised" OR "random" OR "randomly" OR "placebo" OR "assignment")))) AND DOCUMENT TYPES:(Article)	**867**
Embase	1	('familial hypercholesterolemia'/exp OR 'hyperlipoproteinemia type 2':ab,ti) AND 'article'/it	6,696
	2	('cholesterol'/exp OR 'high density lipoprotein cholesterol'/exp OR 'low density lipoprotein cholesterol'/exp OR 'very low density lipoprotein cholesterol'/exp OR 'cholesterol intake'/exp OR 'apolipoprotein'/exp OR 'apolipoprotein a'/exp OR 'apolipoprotein b'/exp) AND [article]/lim	267,296
	3	'intervention studies'/exp OR 'randomized controlled trial'/de OR 'controlled clinical trial'/de OR 'randomized':ab OR 'randomised':ab OR 'randomly':ab OR 'random':ab OR 'controlled trial':ab OR 'interventions':ab OR 'placebo':ab OR 'assignment':ab AND [article]/lim	1,597,142
	4	#1 AND #2	3,688
	5	#1 AND #2 AND #3	**491**

[1] Search date : 2020-11-25.

Table S2: Results of included studies on plasma lipids other than LDL-C, Total-C and Apo B.

Author, Year	Control	Intervention	Outcomes	Post-treatment concentration[1] Control	Post-treatment concentration[1] Intervention	Difference (%)	P value
Food-based interventions							
Gustafsson et al., 1983 [16]	Fully controlled diet with a polyunsaturated-to-saturated fat ratio of 1.3	Fully controlled diet with polyunsaturated-to-saturated fat ratio of 2.0	HDL-C	1.22	1.22	0.0	NS
			VLDL-C	0.42	0.33	-21.4	NS
			LDL-TG	0.55	0.51	-7.3	NS
			HDL-TG	0.22	0.21	-4.5	NS
			VLDL-TG	0.71	0.73	2.8	NS
			Apo A-I	1.11	1.14	2.7	NS
			Apo A-II	1.17	1.18	0.9	NS
Friday et al., 1991 [17]	Fully controlled diet with 82.4 g of butter /2,000 kcal/ day	1. Fully controlled diet with 67 g of safflower oil /2,000 kcal/day	HDL-C	1.27 ± 0.18	1.19 ± 0.16	-6.3	NS
			VLDL-C	0.75 ± 0.15	0.67 ± 0.10	-10.7	NS
			IDL-C	0.67 ± 0.10	0.36 ± 0.08	-46.3	0.02
			TG	1.34 ± 0.29	1.16 ± 0.23	-13.4	NS
			Apo A-1	1.22 ± 0.10	1.30 ± 0.60	6.6	NS
			Apo A-II	0.27 ± 0.02	0.28 ± 0.01	3.7	NS
		2. Fully controlled diet with 67 g of salmon oil/ 2,000 kcal/ day	HDL-C	1.27 ± 0.18	1.11 ± 0.16	-12.6	0.003
			VLDL-C	0.75 ± 0.15	0.21 ± 0.03	-72.0	0.02
			IDL-C	0.67 ± 0.10	0.21 ± 0.05	-68.7	0.01
			TG	1.34 ± 0.29	0.71 ± 0.12	-47.0	0.03
			Apo A-1	1.22 ± 0.10	1.16 ± 0.09	-4.9	NS
			Apo A-II	0.27 ± 0.02	0.24 ± 0.02	-11.1	NS
Laurin et al., 1991 [18]	Fully controlled diet with 35% of protein intake provided from cow's milk	Fully controlled diet with 35% of protein intake provided from soy protein beverage	HDL-C	1.15 ± 0.06	1.20 ± 0.07	4.3	< 0.04
			HDL_2-C	0.55 ± 0.07	0.54 ± 0.07	-1.8	NS
			HDL_3-C	0.60 ± 0.03	0.66 ± 0.02	10.0	<0.03
			VLDL-C	0.45 ± 0.05	0.35 ± 0.04	-22.2	<0.05
			TG	1.02 ± 0.11	0.80 ± 0.08	-21.6	<0.05
			Phospholipids	3.05 ±0.11	2.96 ± 0.09	-3.0	NS
Wolfe et al., 1992 [19]	Fully controlled diet with 10% of energy as protein and 65% as carbohydrate	Fully controlled diet with 27% of energy as protein and 48% as carbohydrate	HDL-C	1.0 ± 0.3	1.1 ± 0.3	10.0	NS
			TG	2.2 ± 0.7	1.7 ± 0.7	-22.7	NS
Fuentes et al., 2008 [20]	Fully controlled diet with 280–300 mg/ day of dietary cholesterol	Fully controlled diet with 150 mg/ day of dietary cholesterol	HDL-C	1.34 ± 0.39	1.37 ± 0.36	2.2	NS
			TG	1.19 ± 0.64	1.14 ± 0.50	-4.2	NS
			Apo A-I	1.44 ± 0.24	1.47 ± 0.26	2.1	NS

Dietary counseling interventions							
Chisholm et al., 1994 [21]	Exchange lists, recipes and nutritional counseling given every 15 or 30 days by an experienced dietician to adopt a high fat diet (38% energy from fat, 14% energy from saturated fat)	Exchange lists, recipes and nutritional counseling given every 15 or 30 days by an experienced dietician to adopt a low fat diet (27% energy from fat, 8% energy from saturated fat)	HDL-C<br>TG	1.44 ± 0.38<br>1.49 ± 0.76	1.33 ± 0.35<br>1.55 ± 0.78	-7.6<br>4.0	<0.05<br>NS
Broekhuizen et al., 2012 [22]	Care as usual (n=159)	Web-based tailored lifestyle advices, 5 personal counselling interventions (1 face-to-face and 4 telephone booster calls); the goal was to adopt and maintain a healthier lifestyle regarding physical activity, saturated fat intake, fruit and vegetables intake, smoking and compliance to statin therapy (n=181)	HDL-C<br>TG	1.2 ± 0.4<br>1.2 ± 0.6	1.2 ± 0.4<br>1.3 ± 0.7	0.0<br>8.3	NS<br>NS
Helk et al., 2019 [23]	Counseling sessions led by an experienced dietician where practical advices on how to replace as many visible fat sources as possible with rapeseed oil were given	Counseling sessions led by an experienced dietician where recipes and practical advices on how to replace as many visible fat sources as possible with rapeseed oil and how to enrich diet in soy protein	HDL-C<br>VLDL-C<br>TG<br>Lp(a)<br>Apo A-I	1.51 ± 0.33<br>0.41 ± 0.09<br>0.90 ± 0.18<br>0.59 ± 0.59<br>1.37 ± 0.15	1.63 ± 0.24<br>0.37 ± 0.12<br>0.81 ± 0.26<br>0.30 ± 0.29<br>1.36 ± 0.13	7.9<br>-9.8<br>-10.0<br>-49.2<br>-0.7	NS<br>NS<br>NS<br>NS<br>NS
Dietary supplement interventions							
ω-3 supplementation							
Balestrieri et al.,1996 [24]	Olive oil capsules (6 g/day)	Fish oil ethyl ester in capsules (6 g/day)	HDL-C<br>TG<br>Apo A-I	1.3 ± 0.5<br>1.3 ± 0.7<br>1.3 ± 0.4	1.4 ± 0.5<br>1.0 ± 0.3<br>1.3 ± 0.5	7.7<br>-23.1<br>0.0	NS<br>NS<br>NS

Chan et al., 2016 [25]	No placebo (usual diet)	ω-3 fatty acid ethyl ester supplement (4 g/day)	HDL-C	1.19 ± 0.12	1.12 ± 0.05	-5.9	NS
			Non-HDL-C	3.39 ± 0.27	3.07 ± 0.18	-9.4	NS
			TG	1.30 ± 0.14	1.05 ± 0.09	-19.2	0.01
			VLDL-apoB-100	0.08 ± 0.01	0.06 ± 0.01	-25.0	0.01
			Apo B-48	0.008 ± 0.002	0.006 ± 0.001	-25.0	0.03
			Lp(a)	0.44 ± 0.11	0.42 ± 0.10	-4.5	NS
Hande et al., 2019 [15]	Olive oil capsules (4 g/day)	ω-3 fatty acid supplements with 460 mg of eicosapentaenoic acid + 380 mg of docosahexaenoic acid (4 g/day)	HDL-C	1.4 ± 0.4	1.4 ± 0.4	0.0	NS
			TG	1.01 ± 0.76	0.74 ± 0.35	-26.7	<0.0001
Phytosterol supplementation							
Gylling et al., 1995 [26]	Rapeseed oil margarine without sitostanol ester	Rapeseed oil margarine enriched in sitostanol ester (3 g/day of sitostanol)	HDL-C	1.20 ± 0.07	1.25 ± 0.08	4.2	NS
			HDL_2-C	0.62 ± 0.08	0.68 ± 0.08	9.7	NS
			HDL_3-C	0.51 ± 0.02	0.50 ± 0.02	-2.0	NS
			VLDL-C	0.26 ± 0.06	0.25 ± 0.07	-3.8	NS
			IDL-C	0.27 ± 0.08	0.20 ± 0.07	-25.9	<0.05
			HDL-C/LDL-C	0.23 ± 0.03	0.30 ± 0.04	30.4	<0.05
			TG	1.03 ± 0.13	0.92 ± 0.12	-10.7	NS
			Phospholipids	3.16 ± 0.11	2.94 ± 0.11	-7.0	<0.05
Neil et al., 2001 [27]	Placebo fat spread (25 g/day)	Plant sterol-enriched fat spread (25 g/day) providing 2.5 g/day of phytosterols (β-sitosterol: 50%; campesterol: 25%; stigmasterol: 20%; other sterols: 5%)	HDL-C	1.43 ± 0.36	1.49 ± 0.36	4.2	<0.01
			VLDL-C	0.81 ± 0.38	0.73 ± 0.30	-9.9	NS
			TG	1.29 (95% CI: 0.66-3.93)	1.27 (5% CI: 0.65-3.80)	-1.6	NS
			Apo A-I	1.47 ± 0.26	1.41± 0.25	-4.1	NS
Amundsen et al., 2002 [28]	Placebo fat spread (20 g/day)	Plant sterol-enriched fat spread (20 g/day) providing 1.60 ± 0.13 g / day of sterol esters (sitosterol: 50%)	HDL-C	1.25 ± 0.31	1.26 ± 0.35	0.8	NS
			TG	0.78 ± 0.33	0.80 ± 0.37	2.6	NS
			Apo A-I	1.35 ± 0.23	1.32 ± 0.26	-2.2	NS
de Jonhg et al., 2003 [29]	Placebo margarine (15 g/day)	Plant sterol spread (15 g/day) providing 2.3 g of sterols (sitosterol: 46.9%; campesterol: 27.3%;	HDL-C	1.29 ± 0.29	1.31 ± 0.31	1.6	NS
			TG	0.90 ± 0.40	0.85 ± 0.36	-5.6	NS

		stigmasterol: 16.3%; other sterols: 9.5%)					
O'Neill et al., 2004 [30]	Placebo margarine (20 g/day)	1. Plant sterol-enriched fat spread (20 g/day) providing 1.6 g / day of free sterols + 1 placebo cereal bar (25 g/day)	HDL-C	1.33 ± 0.06	1.31 ± 0.07	-1.5	NS
			Total-C/HDL-C	4.6 ± 0.20	4.6 ± 0.22	0.0	0.05
			TG	1.43 ± 0.09	1.31 ± 0.08	-8.4	NS
		2. Plant stanol-enriched fat spread (20 g/day) providing 1.6 g / day of free stanols + 1 placebo cereal bar (25 g/day)	HDL-C	1.38 ± 0.06	1.33 ± 0.05	-3.6	NS
			Total-C/HDL-C	4.4 ± 0.18	4.3 ± 0.18	-2.3	NS
			TG	1.43 ± 0.12	1.32 ± 0.13	-7.7	NS
		3. Plant stanol-enriched fat spread (20 g/day) providing 1.6 g/day of free stanols + 1 cereal bar (25 g/day) providing 1.0 g/day of stanol ester	HDL-C	1.30 ± 0.05	1.24 ± 0.05	-4.6	0.02
			Total-C/HDL-C	4.6 ± 0.20	4.4 ±0 .20	-4.3	0.03
			TG	1.60 ± 0.09	1.36 ± 0.09	-15.0	0.005
Jakulj et al., 2006 [31]	Low-fat placebo yogurt (500 mL/day)	Low-fat yogurt enriched with 2.0 g of plant stanols (500 mL/day)	HDL-C	1.38 ± 0.27	1.35 ± 0.24	-2.2	NS
			TG	0.57 (95% CI: 0.51 - 0.93)	0.61 (95% CI: 0.51-0.84)	7.0	NS
Fuentes et al., 2008 [20]	Fully controlled diet with 280–300 mg/day of dietary cholesterol and 0.5 g/day of plant sterols	Fully controlled diet with 280–300 mg/day of cholesterol and 2.5 g/day of plant sterols	HDL-C	1.34 ± 0.39	1.37 ± 0.31	2.2	NS
			TG	1.19 ± 0.64	1.07 ± 0.52	-10.1	NS
			Apo A-I	1.44 ± 0.24	1.47 ± 0.21	2.1	NS
Other type of dietary supplement							
Wirth et al., 1982 [32]	Usual diet	Granulated guar gum form (15.6 g/day)	HDL-C	1.42 ± 0.39	1.24 ± 0.34	-12.7	NS
			LDL-C/HDL-C	4.96	5.20	4.8	NS
			TG	1.47 ± 0.79	1.87 ± 0.50	27.2	NS
			Apo A-I	1.17 ± 0.12	1.21 ± 0.10	3.4	NS
Greyling et al., 2006 [33]	Placebo capsules	Policosanol supplements providing 20 mg of policosanol / day	HDL-C	1.19 (95% CI:1.06 - 1.32)	1.25 (95% CI: 1.08-1.41)	5.0	NS
			TG	1.32 (95% CI: 0.93 - 1.71)	1.29 (95% CI: 0.89 - 1.68)	-2.3	NS

[1] Values are expressed in mmol/L for lipoprotein fractions and in g/L for apolipoproteins.
